# Video Endoscopic Inguinal Lymphadenectomy (VEIL) -a prospective critical perioperative assessment of feasibility and morbidity with points of technique in penile carcinoma

**DOI:** 10.1186/1477-7819-11-42

**Published:** 2013-02-22

**Authors:** Harvinder Singh Pahwa, Sanjeev Misra, Awanish Kumar, Vijay Kumar, Akash Agarwal, Rohit Srivastava

**Affiliations:** 1 Department of Surgery, CSM Medical University (Formerly King George’s Medical University), 226003, Lucknow UP, India; 2 Department of Surgical Oncology, CSM Medical University (Formerly King George’s Medical University), 226003, Lucknow UP, India; 3 New Surgical Block, Department Of Surgery, C.S.M.U. (Erst. K.G.M.U.), 226005, Lucknow UP, India

**Keywords:** Penile cancer, Inguinal lymphadenectomy, Video-assisted surgery, Video endoscopic inguinal lymphadenectomy (VEIL)

## Abstract

**Background:**

Inguinal lymph node involvement is an important prognostic factor in penile cancer. Inguinal lymph node dissection allows staging and treatment of inguinal nodal disease. However, it causes morbidity and is associated with complications, such as lymphocele, skin loss and infection. Video Endoscopic Inguinal Lymphadenectomy (VEIL) is an endoscopic procedure, and it seems to be a new and attractive approach duplicating the standard open procedure with less morbidity. We present here a critical perioperative assessment with points of technique.

**Methods:**

Ten patients with moderate to high grade penile carcinoma with clinically negative inguinal lymph nodes were subjected to elective VEIL. VEIL was done in standard surgical steps. Perioperative parameters were assessed that is - duration of the surgery, lymph-related complications, time until drain removal, lymph node yield, surgical emphysema and histopathological positivity of lymph nodes.

**Results:**

Operative time for VEIL was 120 to180 minutes. Lymph node yield was 7 to 12 lymph nodes. No skin related complications were seen with VEIL. Lymph related complications, that is, lymphocele, were seen in only two patients. The suction drain was removed after four to eight days (mean 5.1). Overall morbidity was 20% with VEIL.

**Conclusion:**

In our early experience, VEIL was a safe and feasible technique in patients with penile carcinoma with non palpable inguinal lymph nodes. It allows the removal of inguinal lymph nodes within the same limits as in conventional surgical dissection and potentially reduces surgical morbidity.

## Background

Penile carcinoma is an important health problem in several developing countries, including India [1]. Inguinal lymph node involvement is an important cause of morbidity and an important predictive factor for survival in penile cancer patients [2-4]. In high risk patients, elective inguinal lymphadenectomy may offer survival advantage over watchful waiting [5,6]. Elective inguinal lymphadenectomy is the standard of care for patients with larger tumour size, high histological grade and the presence of lymphovascular invasion [1,6,7]. Studies show that conventional inguinal lymph node dissection is associated with major complications such as lymphocele, skin loss and infection [8]. Some authors have described alternative procedures to reduce the morbidity of the treatment of inguinal lymph nodes, mainly by decreasing the area of dissection [9-11] but oncological results may not be as good as those with the radical procedure [12,13].

An endoscopic procedure, with small incisions away from the dissecting area, seems to be a new and attractive approach, duplicating the standard open procedure with less morbidity [14]. We describe here points of technique and perioperative outcome with Video Endoscopic Inguinal Lymphadenectomy (VEIL) in patients with carcinoma of the penis.

## Methods

Ten patients with locally advanced and/or high grade squamous cell carcinoma of the penis with no palpable inguinal lymph nodes suitable for elective inguinal lymph node dissection were enrolled in this study. The mean age of the patients was 51 years (range 39 to 62 years). The majority (70%) of patients were at stage T2 while those at stages T1 and T3 were 20% and 10%, respectively. In majority (60%) of the patients, the tumor was moderately differentiated, whereas in 40%, it was poorly differentiated. Partial penectomy was done in 70% of patients while 30% underwent total penectomy.

All patients underwent standard inguinal lymph node dissection, sparing the saphenous vein by VEIL. The techniques and surgical steps of VEIL are described below. The perioperative parameters assessed were the duration of surgery, lymph-related complications, time to drain removal, lymph node yield, surgical emphysema and histopathological positivity of lymph nodes.

This procedure is being carried out in our institute as a routine procedure and it was not done as a part of experimental research work so ethical clearance was not required.

### Consent

Written informed consent was obtained from all the patients for publication of this report and any accompanying images.

### Surgical steps of VEIL

We used the same technique as used for standard inguinal lymph node dissection.

The aim of this approach was to remove all of the lymph nodes that were at the most probable locations for first-line lymphatic invasion. Our technique for creation of space was based on the technique described by Tobias-Machado et al. [14].

**Figure 1:**
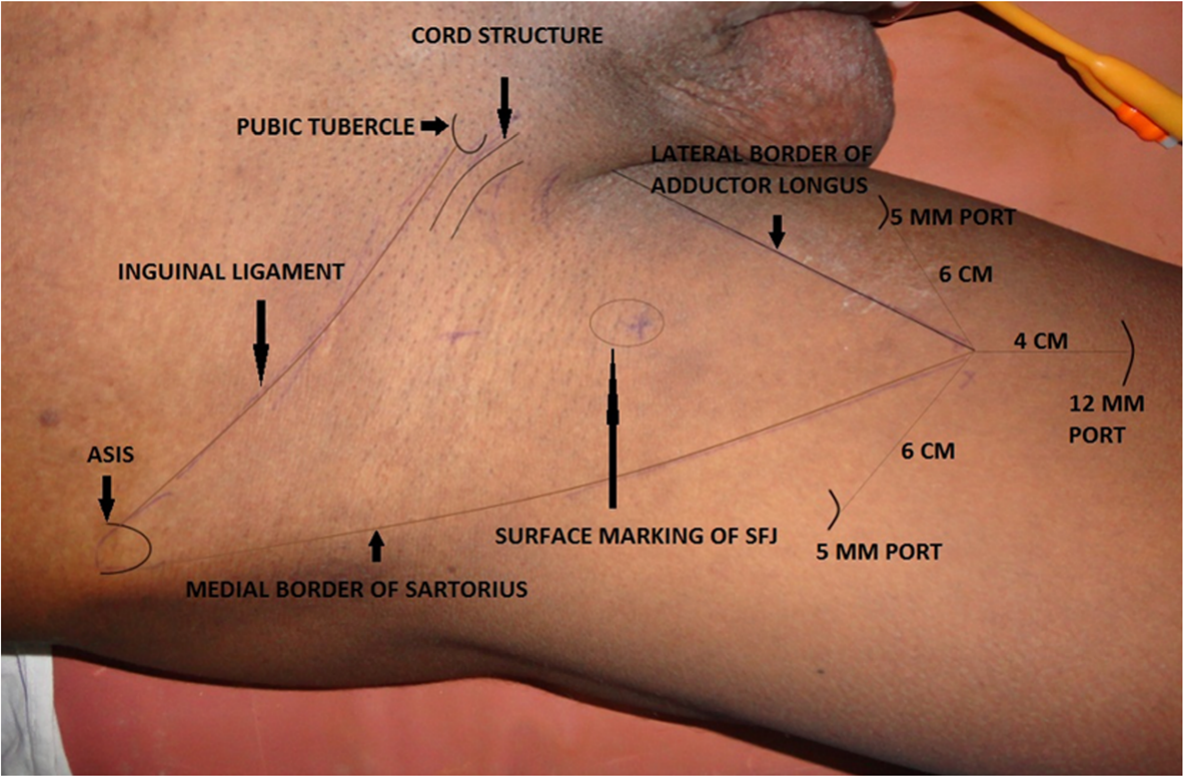
Surface marking showing important land marks.

**Figure 2:**
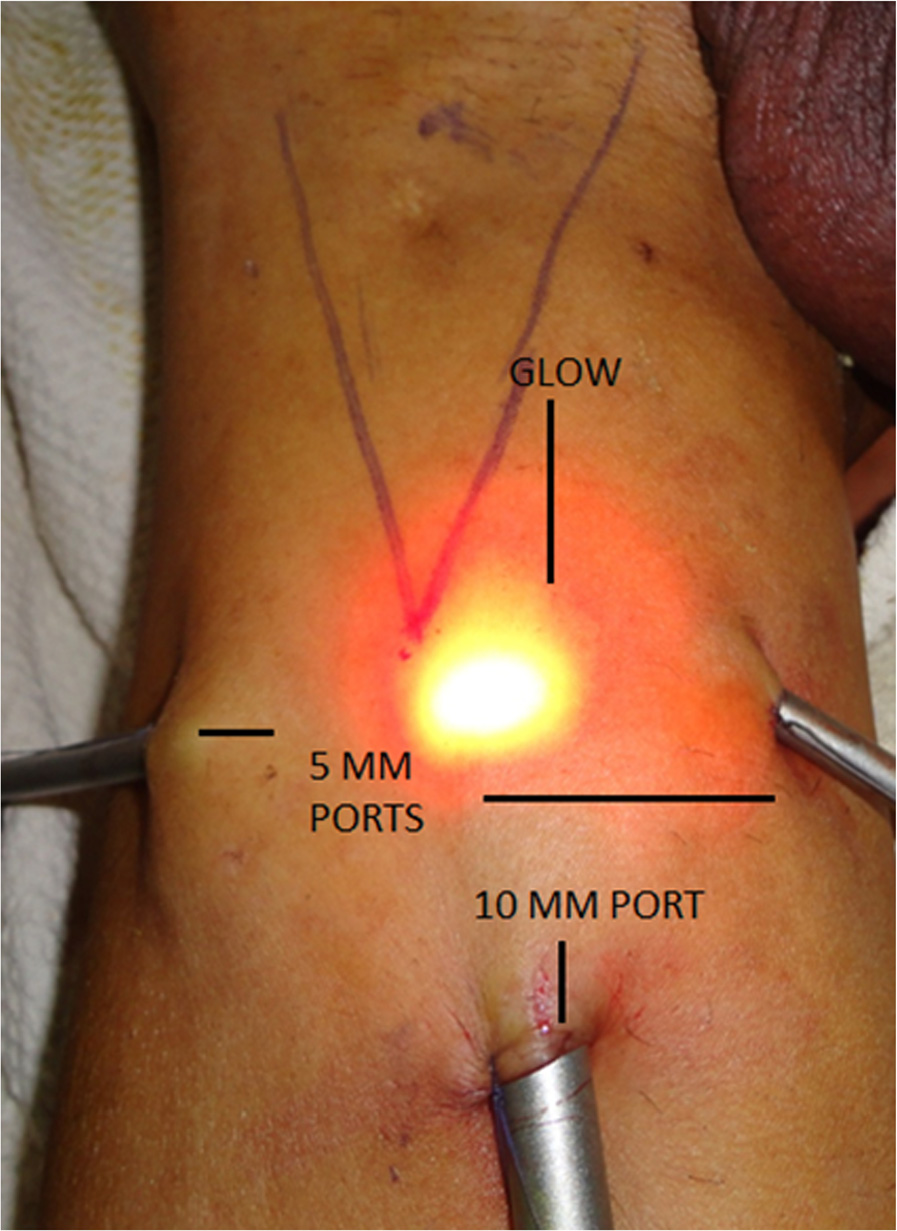
Port Placement and illuminated skin.

**Figure 3:**
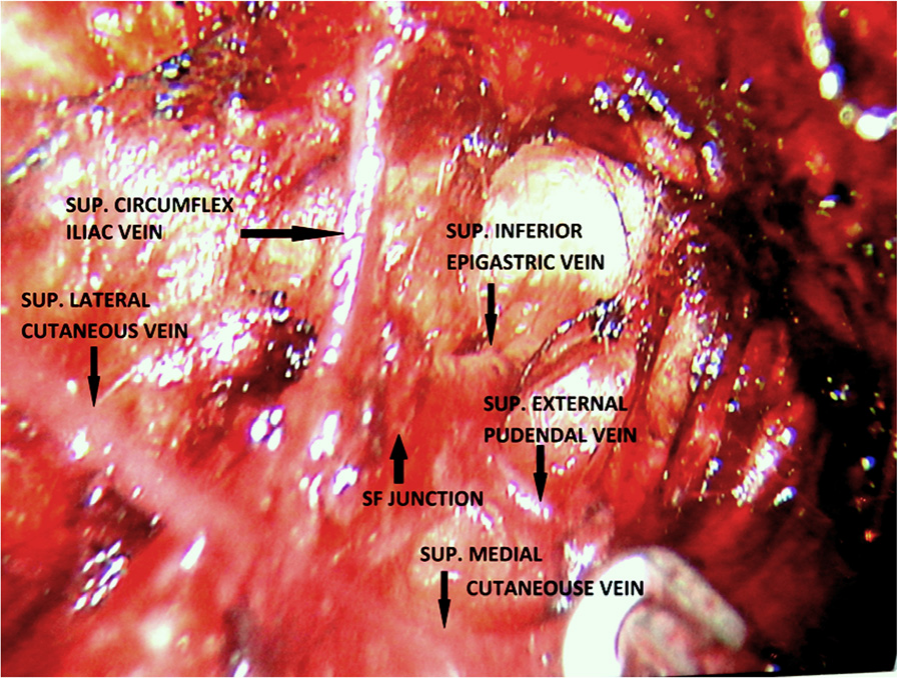
Saphenofemoral junction and Great saphenous vein with its tributaries.

**Figure 4:**
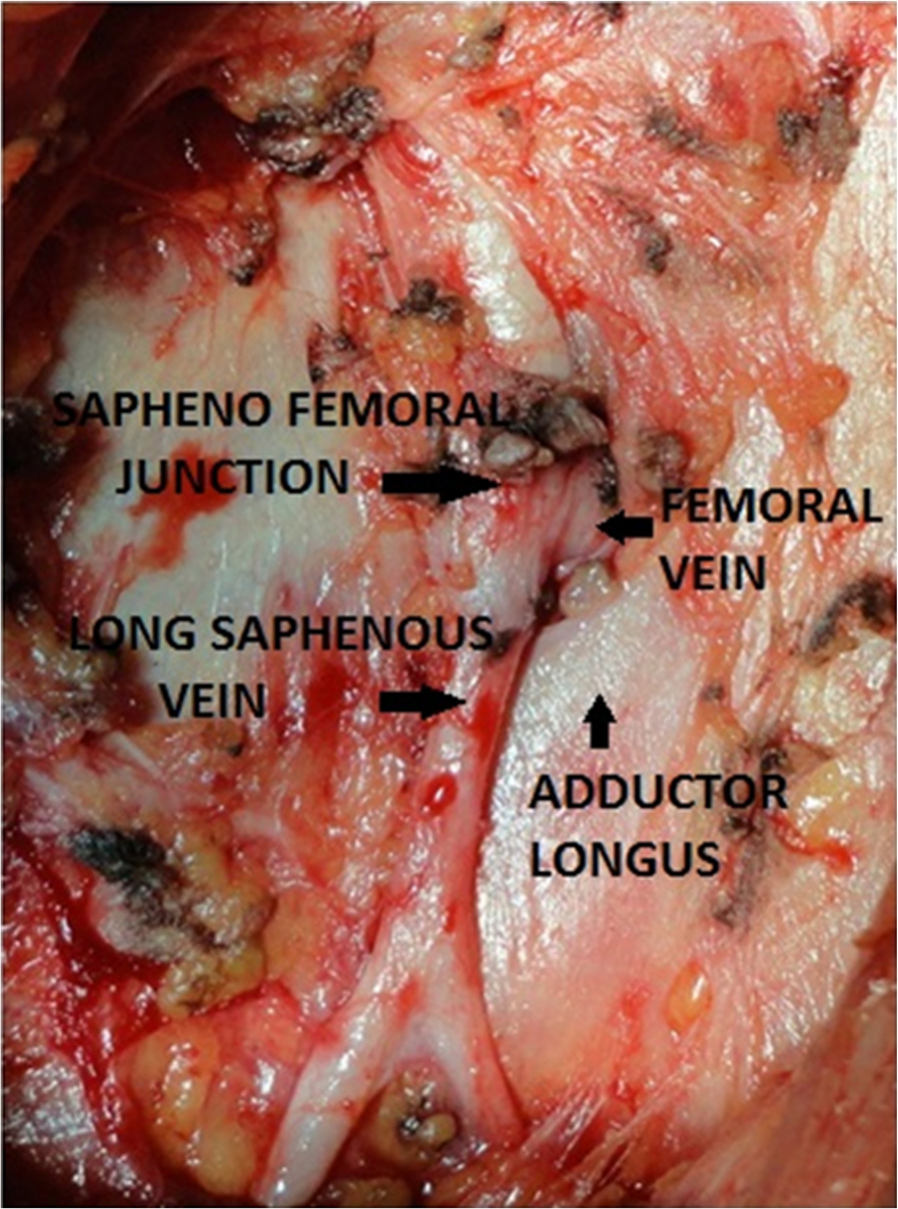
Saphenofemoral junction - final view after complete clearance.

1. Patient positioning-. After epidural block, the patient was positioned in the same way as in open inguinal lymph node dissection. The surgeon and assistant were positioned laterally to the patient’s leg on the side of the operation and the video monitor system was placed at the opposite side next to the patient’s waist.

2. Surface marking was done for the spermatic cord, inguinal ligament, anterior superior iliac spine, sapheno-femoral junction and femoral triangle (Figure 1).

3. Port placement-. A 12-mm incision was made 4 cm distally to the lower vertex of the femoral triangle. Initially, scissors and dissecting forceps were used to develop a plane of dissection deep to the Scarpa’s fascia. The second and third 5-mm ports were placed 6 cm superomedially and superolaterally to the apex of the triangle (Figure 2). Trocars were fixed with sutures. The first port accommodated a zero degree telescope.

4. Gas insufflation-. The working space was insufflated with CO_2_ at 12 mmHg with quick space distension.

5. Retrograde dissection and identification of anatomical limits-. The dissection was carried out deep to the Scarpa’s fascia and superiorly, up to the external oblique fascia, so that all superficial lymphatic tissue could be removed. The main landmarks of dissection were medially the adductor longus muscle, laterally - the sartorius muscle, superiorly - the external oblique aponeurosis above the level of spermatic cord, and the inferior margin was the apex of the femoral triangle. Trans illumination, external pressure on skin and surface markings allow good orientation and monitoring of the progression of the dissection area towards the cavity (Figure 2).

6. Identification of landmarks-. The saphenous vein was identified medially and the spermatic cord and the external inguinal ring, superomedially. The branches of the femoral nerve, present laterally, were identified and preserved. The identified saphenous vein was dissected cranially up to the fossa ovalis.

7. The femoral artery was identified at the femoral triangle. At this point the muscular fascia was opened in all its extension.

8. Distal lymphatic tissue was coagulated with harmonic scalpel and cut at the vertex of the femoral triangle. Lymphatic tissue dissection was carried upwards along the saphenous vein till we reached the femoral vessels above the femoral ring.

9. Branches of the saphenous vein, namely, the superficial circumflex, superficial epigastric, superficial external pudendal, superficial lateral and medial cutaneous (Figure 3), were safely managed using harmonic and bipolar cautery and, as a result, no clips were used. Dissection of lympho-fatty tissue was completed (Figure 4).

10. A complete inguinal dissection was carried out as mentioned previously. Dissection ended by liberating the specimen consisting of fibrofatty tissue along with inguinal lymph nodes after coagulation of the proximal part of the lymphatic tissue with harmonic scalpel at the deep portion of the femoral channel.

11. The specimen having inguinal lymph nodes was removed via canula through the first 12-mm port. If the specimen was larger the incision was increased and a larger canula or retrieval bag was used for removal of the specimen.

12. Port incisions were closed after placement of suction drainage through the lateral port.

13. An elastic compression bandage was applied from the lower part of leg to the thigh and early mobilization with lower limb physiotherapy was practiced. The drain was removed after four to eight days once drainage volume was less than 50 ml in 24 hours.

## Results

The intraoperative and postoperative period was uneventful in all the surgical patients. Operative time for VEIL was 120 to 180 minutes. Total operative time for VEIL was initially longer but was reduced subsequently (Table 1). No skin-related complications were seen. Lymph-related complication, a lymphocele, was seen in 2/10 (20%) patients. This was managed by aspiration with a needle (single aspiration in one patient and two aspirations in the second patient). The suction drains were removed after four to eight days (mean 5.1 days). Lymph node yield was 7 to 12 lymph nodes. Self-resolving (one to three days) infra-umblical surgical emphysema was observed in all the patients. Only one patient had two positive lymph nodes on histopathology, so none of our patients required pelvic lymph node dissection. In our initial experience with VEIL, the overall morbidity was only 20%. We present here our perioperative results, although we have had a short follow-up of 3 to 14 months.

**Table 1:** Perioperative outcome of patients with squamous cell carcinoma of the penis undergoing VEIL

**SN of patients**	**Duration of surgery (in minutes)**	**Skin-related complications**	**Lymph-related complications**	**Drain removal (in days)**	**Lymph node yield**	**Self-resolving infra-umblical surgical emphysema**	**Histopathology of lymph nodes (positive/negative for malignancy)**
1	170	No	No	5	8	Positive	Negative
2	180	No	No	5	7	Positive	Negative
3	175	No	Lymphocele	8	12	Positive	Negative
4	160	No	No	6	11	Positive	Negative
5	120	No	No	5	14	Positive	Negative
6	130	No	No	5	10	Positive	Positive (2)
7	140	No	Lymphocele	7	10	Positive	Negative
8	120	No	No	7	12	Positive	Negative
9	125	No	No	4	12	Positive	Negative
10	120	No	No	4	10	Positive	Negative

## Discussion

Inguinal lymph node involvement is present in approximately 30% of patients with clinically negative nodes and it is an important cause of morbidity and an important predictive factor for overall survival in penile cancer patients [3,4]. In high risk patients with penile cancers, studies have shown that elective inguinal lymphadenectomy offers better survival than salvage lymphadenectomy [1,6,7]. However, conventional inguinal lymph node dissection is associated with major complications, such as lymphocele, skin loss and infection [8]. As the morbidity associated with surgery is high, its role is being questioned, especially when the intention is prophylactic. Various techniques have been tried to reduce morbidity by reducing inguinal dissection templates [9-11,15,16] or by doing sentinel lymph node biopsy with a radioisotope [17]. The VEIL technique was described by Tobias-Machado et al. in 2006[14] with an aim to duplicate the standard radical procedure with less morbidity. By using VEIL, we were able to identify the same landmarks of the open surgery and perform a dissection following the same template of the radical surgery. We can see clearly through the endoscopic view if all lymphatic tissue within the limits of dissection has been removed at the end of the procedure as is the case in open surgery. We were able to perform complete inguinal lymph node dissection in all our patients with this technique. We have selected patients with clinically negative groins as this was our initial experience. This procedure has also been used for clinically positive inguinal nodes but has not been investigated in patients with bulky inguinal nodes. Pelvic lymph node dissection as it is only required if more than two nodes are positive on histopathology in clinically N0 lymph nodes [18], so none of our cases required pelvic lymph node dissection.

Complications with VEIL are fewer compared to an open surgical procedure, and this technique has the potential to reduce post-operative morbidity. The most important advantage of VEIL seems to be a decrease in skin events and, in our experience, there were no skin related complications. Tobias- Machado et al. reported 0% cutaneous and 20% overall morbidity [19], whereas Sotelo et al. reported 23% lymphatic morbidity in their study [20]. Similar experience has been reported from other studies [14,21]. With VEIL the drains can be removed sooner and patients can be discharged earlier [10]. Operative time of VEIL was longer than with conventional open surgery, but the time decreased significantly as our experience with the procedure grew. As the incisions are small in VEIL, the results are more aesthetically pleasing. Master et al. [22] reported their experience with 25 groin dissections performed endoscopically. They performed complete inguinofemoral lymphadenectomy (Leg endoscopic groin lymphadenectomy − the LEG procedure) and stated that this procedure required operating time comparable to open procedures and carried less morbidity.

There are few reports in the literature of robotic-assisted video endoscopic inguinal node dissection. The first such case was reported by Josephson et al. [23] in 2009. They performed endoscopic robotic-assisted inguinal lymph node dissection in a patient with penile cancer. Dogra et al. [24] published their experience with two cases of robotic-assisted inguinal node dissection in patients with carcinoma of the penis in 2011.

There may be apprehension regarding potential risk of tumor seeding due to infra-umbilical emphysema. Although there are no studies in this regard for video endoscopic inguinal lymph node dissection, there are studies evaluating the role of pneumoperitoneum in tumour seeding in laparoscopic surgeries. In human and animal studies, CO2 was not able to aerosolize large numbers of tumor cells using pressures of 8 to 15 mmHg [25]. Further studies with long term follow-up are required to dispel the doubt regarding the role of infra-umbilical emphysema in tumur seeding.

New studies with a greater number of patients and long-term follow-up are needed to test the hypothesis that VEIL can retain the long-term oncological efficacy of the standard surgery and result in a lower morbidity. If this holds true, VEIL can become an attractive choice for the prophylactic inguinal lymphadenectomy in penile cancer patients. Other possible clinical indications for this new procedure may include prophylactic dissection for urethral and vulval cancers.

## Conclusion

Our preliminary results show that video endoscopic inguinal lymphadenectomy is a safe and feasible technique in patients with penile carcinoma with no palpable lymph nodes. It allows the radical removal of inguinal lymph nodes within the same limits of conventional surgical dissection and potentially reduces surgical morbidity. VEIL has the potential to become the minimally invasive procedure for low volume inguinal lymph node disease and prophylactic inguinal lymph node dissection but long term studies with a greater number of patients are needed.

## Abbreviations

VEIL: Video endoscopic inguinal lymphadenectomy

## Competing interests

All authors declare that they have no competing interests.

## Authors’ contributions

HSP was the operating surgeon and made substantive intellectual contributions in this study, including acquisition of data, interpretation and drafting/editing of the manuscript, and has given final approval of the version to be published. SM has given substantive intellectual contributions in this study and helped in the interpretation and drafting/editing of the manuscript, and has given final approval of the version to be published. AK, VK, AA, and RS made substantive contributions to this study, including acquisition of data, interpretation and drafting/editing of the manuscript, and have given final approval of the version to be published. All authors read and approved the final manuscript.
